# An inexpensive retrospective standard setting method based on item facilities

**DOI:** 10.1186/s12909-020-02418-5

**Published:** 2021-01-06

**Authors:** John C. McLachlan, K. Alex Robertson, Bridget Weller, Marina Sawdon

**Affiliations:** 1 University of Central Lancashire, Harrington Building, 11 Victoria St, Preston, PR1 7QS UK; 2 CNTW Trust, Hopewood Park Hospital, Ryhope, Sunderland, SR2 0NB UK; 3 School of Medicine, University of Sunderland, Chester Rd, Sunderland, SR1 3SD UK

**Keywords:** Standard-setting, Retrospective, Cost, Rapid, Exponential

## Abstract

**Background:**

Standard setting is one of the most challenging aspects of assessment in high-stakes healthcare settings. The Angoff methodology is widely used, but poses a number of challenges, including conceptualisation of the just-passing candidate, and the time-cost of implementing the method. Cohen methodologies are inexpensive and rapid but rely on the performance of an individual candidate. A new method of standard setting, based on the entire cohort and every item, would be valuable.

**Methods:**

We identified Borderline candidates by reviewing their performance across all assessments in an academic year. We plotted the item scores of the Borderline candidates in comparison with Facility for the whole cohort and fitted curves to the resulting distribution.

**Results:**

It is observed that for any given Item, an equation of the form

y ≈ C. e^Fx^

where y is the Facility of Borderline candidates on that Item, x is the observed Item Facility of the whole cohort, and C and F are constants, predicts the probable Facility for Borderline candidates over the test, in other words, the cut score for Borderline candidates. We describe ways of estimating C and F in any given circumstance, and suggest typical values arising from this particular study: that C = 12.3 and F = 0.021.

**Conclusions:**

C and F are relatively stable, and that the equation

y = 12.3. e^0.021x^

can rapidly be applied to the item Facility for every item. The average value represents the cut score for the assessment as a whole. This represents a novel retrospective method based on test takers.

Compared to the Cohen method which draws on one score and one candidate, this method draws on all items and candidates in a test. We propose that it can be used to standard set a whole test, or a particular item where the predicted Angoff score is very different from the observed Facility.

## Background

Standard setting is both important and problematic in medical education. The Angoff method [1] is widely used for standard setting selected-response items in high stakes settings such as the General Medical Council tests for non-UK, non-EU doctors wishing to practice in the UK, and USMLE Step 1, yet its use poses a number of challenges.

Perhaps the most significant of these is the requirement that assessors conceptualise a particular kind of candidate, often described as the ‘minimally competent’ or ‘Borderline’ candidate. In the context of Angoff standard setting, ‘Borderline’ generally represents a ‘Borderline pass’, and it is in this sense that we use it here.

Whichever form of words is used, assessors may have very different ideas of what that class of candidates represents. This is compounded by the fact that subject specialists among the assessors may lack generalist knowledge [2], or lack awareness of what particular level candidates would appropriately have achieved.

As a consequence, a minimum number of assessors may be required, and this in itself poses practical problems in identifying a sufficient number of assessors with sufficient expertise in the subject, and indeed experience in using the Angoff method. One safety-net option is to use the Hofstee compromise method [3] if any ‘Angoffed’ assessment fails a ‘Reality Check’ [4].

A particular tendency of novice assessors is ‘reversion to the mean’, where they tend to award Angoff scores of around 50% rather than using the full scale range. This results in a low correlation between the predicted Angoff value and the observed Facility (where Facility is the percentage of candidates answering correctly) of the items.

Some of the same considerations apply to Ebel standard setting [5]. Again, the just-passing candidate is difficult to conceptualise, and a panel of experts is required to carry out the required classification.

An inexpensive alternative is to use either the Cohen method [6], which derives the cut score from a multiple of the 95th centile candidate, or the similar modified Cohen method [7], which relies on the 90th centile candidate. These methods are quick to implement, and do not require the input of expensive staff time. However, they may be criticised on the basis that they rely on the score of an individual candidate (or in the case of ties, a small number of candidates). We return to this issue in the Discussion.

However, it is possible that assessments vary more in difficulty than does the ability of the cohort, since medical students are highly selected for academic ability prior to entry. In this case, the difficulty of the assessment may be the key variable, and the cumulative Facility of the items is a guide to this.

Of course, Facility represents the whole cohort performance, rather than the performance of the Borderline candidates. We hypothesised that for good quality One-Best-of-Five MCQs, the relationship between Facility for the whole cohort, and the Facility for Borderline candidates, would be curvilinear in nature, with the difference between them approaching zero as the Facility approaches 100 and 20%. This is because if the entire cohort scores an item correctly, then so will the Borderline candidates, and if the best candidates do no better than guessing, then neither will the Borderline candidates.

In this study we therefore attempted to explore the effect of classifying different numbers of students as ‘Borderline’ in comparison with the cohort as a whole. Classification was carried out based on performance across the whole range of modules undertaken by the students as described in the Methods.

The exact nature of the relationship between whole cohort and Borderline Facility will depend on the proportion of Borderline candidates in the class, and we discuss ways in which this might be estimated.

Where such a relationship emerges, it would be of value in assisting novice Angoff assessors in estimating the performance of Borderline candidates for an item which had been used before. It could also be used for adjusting any items where the discrepancy between the predicted Angoff value and the observed Facility for that item is greater than seems plausible.

More importantly, the relationship could be used by itself as a standard setting method in conditions in which Angoff or similar methods were not practical: for instance, if too few subject matter experts were available to form an assessor panel, or where the resource costs of using the Angoff method were too high. This would then be a retrospective method based on test takers, rather than a prospective method based on test items.

The purpose of this study is to show proof of concept and although the analyses were carried out locally, we believe our results would be adaptable and of interest to other settings outside our school.

## Methods

The analyses were based on a cohort of students at a UK Medical School. The number of students involved was in the region of one hundred, but the exact number is not disclosed since this may enable the particular cohort to be identified. Student names were never used in the analysis, and student numbers were re-coded automatically so anonymity was preserved. The data were used retrospectively, and this analysis has played no part in summative decisions.

All calculations were carried out, and graphs plotted, using Microsoft Excel©.

Ethical approval for the project on this basis and for publication of results was granted by the relevant University Ethical Approval Committee (approval code STEMH 1058).

The First Year medical student course in question contains three modules each year. Modules 1 and 2 address declarative knowledge, and contained a total of three papers, and Module 3 involves an OSCE skills assessment. Standards are set for Modules 1 and 2 by the modified Angoff method, and for Module 3 by Borderline Regression. Module 3 had an additional conjunctive condition which was that candidates had to pass at least 75% of the OSCE stations.

The anonymised candidates were classified by their performance in each of their modules, with reference to the Standard Error of Measurement (SEM) of the exam and given a corresponding score as described in Table 1.

**Table 1:** Boundaries and score allocations for various Borderline categories

Description	Boundaries	Score
Possible Borderline	between 1 and 2 SEM *above* the cut score	0.5 points
Probable Borderline	within 1 SEM of the cut score	1 point
Definite Borderline	between 1 and 2 SEM *below* the cut score	2 points

For the skills module, candidates who had failed 25% of the OSCE stations were also considered Borderline and scored 1 point. See Table 2 for the distribution of scores in this particular cohort.

**Table 2:** Proportions of candidates scoring various numbers of ‘borderline’ points as calculated in the text. Those scoring 0.5 points lay between 1 and 2 standard errors of measurement above the cut score

‘Borderline’ Points	% of Cohort
0.5	12
1	9
1.5	5
2	4
2.5	2
3	2
3.5	1
4	1
4.5	2
5	2
5.5	0
6	0
6.5	2
All	43

Obviously, a candidate could gather points from more than one module. Points ranged from 0.5 for approximately 12% of the cohort, to 6.5 for a few individuals. In total, approximately 43% of the cohort had points. However, a total score of 0.5 points represented a performance between one and two SEM *above* the cut score in one Module only, which is likely to be the result of chance for an otherwise satisfactory candidate.

The Facility of the Borderline candidates for each item was plotted against the cohort facility, first for all Borderline candidates, then for a variety of different score combinations. Curves were fitted to these plots using the trendline function in Excel. This allowed us to explore the stability of the curve in terms of it’s constants.

A standardised ‘exponential curve’ showing the relationship between the Facility of the Borderline candidates and that of the cohort as a whole was then developed. It was retrospectively applied to a total of 26 previous MCQ-style assessments over the last 4 years of the Undergraduate medical programme as a standard setting method. Cut scores were calculated on the basis of this exponential curve and compared to those which had been obtained by a full Angoff procedure. Cohen and Modified Cohen method cut scores were also calculated for each exam, although in practice only Angoff methods had been used. From these, the proportion of candidates who would have failed each assessment by each method were calculated. These results were plotted against the average score in each assessment.

A further theoretical calculation showing the effect of varying the proportion of candidates classed as Borderline in the cohort was also carried out.

## Results

**Fig. 1:**
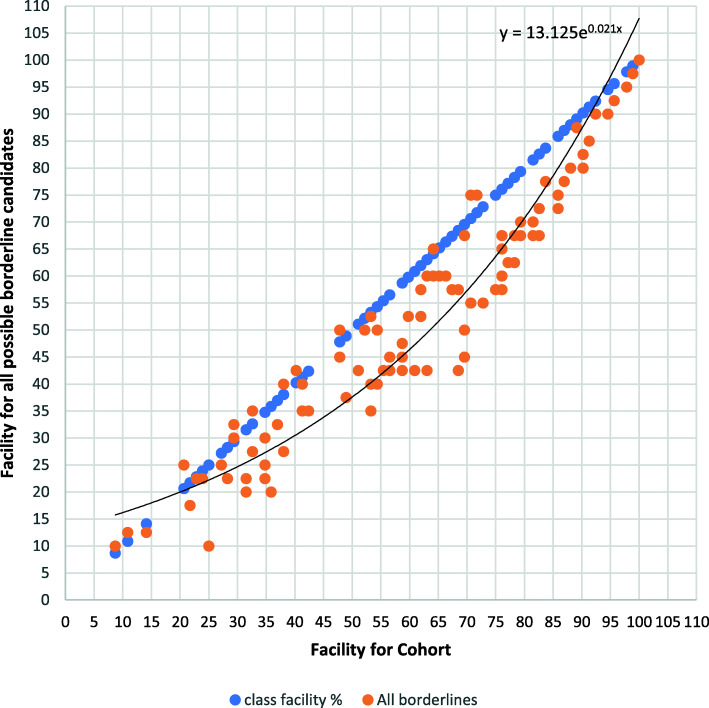
Facility for all candidates plotted against all possible borderline candidates. As a reference, cohort Facility is plotted against itself as a 45° slope

A plot was constructed of the Facility of (a) (shown in Table 3) each Item in the test compared to the score of all Borderline candidates, and the trendline added (Fig. 1). As can be seen, as predicted a curved trendline, approaching zero at Item Facilities of 0 and 100 is indeed observed. The equation for this curve is shown on Fig. 1, and is of the form

$$ \mathrm{y}\approx \mathrm{C}.{\mathrm{e}}^{\mathrm{Fx}} $$

**Table 3:** The values observed for curves of the form of Eq. 2. A family of curves could be selected for the ‘Standard’ values; this particular combination was chosen because the difference from the Facility is zero at 20 and 100%

	Range of Borderline scores	% of cohort	C	F
(a)	All possible Borderlines	43	13.125	0.021
(b)	1–6.5	32	12.756	0.0208
(c)	1.5–6.5	23	13.562	0.0192
(d)	1–5.5	27	12.6	0.0218
(e)	0.5–6.5 (Excluding Source)	28	12.964	0.0209
Exponential			12.3	0.021

Where y is the Facility of Borderline candidates, x is the observed Facility of the cohort as a whole, and C and F are constants.

This process was repeated for various combinations of possible Borderline candidates, to explore how stable this curve was in terms of its constants. As listed in Table 3, these combinations were (b) excluding those who had scored only 0.5 points (i.e. had scored between 1 and 6.5 points) on the basis that a score of 0.5 (between 1 and 2 SEM above the cut-score in a single module) probably represents noise in the performance of otherwise capable students (c) students who fell between 1.5 and 6.5 points, a more stringent interpretation of Borderline (d) candidates scoring between 1 and 5.5 points (excluding those candidates who would be clear fails and (e) showing only scores on different assessments from that shown in the plot, so that there is no element of circularity in the reasoning. The results are shown in Table 3.

As can be seen, these curves are all relatively consistent in terms of their constants. On this basis, a standard exponential curve was calculated on the basis that it intercepts Facility exactly at 20 and 100%. This curve had the constant values

y = 12.3e^0.021x^

This equation can therefore be applied to the Facility of any individual item in a test and gives the expected score for a Borderline candidate for that item. The average of these values is therefore the cut score for the test as a whole.

For the 26 assessments over the four-year period of this study, the proportion of candidates who would have failed each assessment by Angoff, Cohen, Modified Cohen and use of the exponential equation were calculated. Average values for these are shown in Table 4.

**Table 4:** Percentage of ‘Fail’ students over a total of 26 exams, using 4 different standard setting methods

	Angoff	Exponential	Cohen	Modified Cohen
Mean	15.13484	14.08984	25.36721	13.39841
Standard Deviation	9.528302	5.759773	10.97905	7.06995

**Fig. 2:**
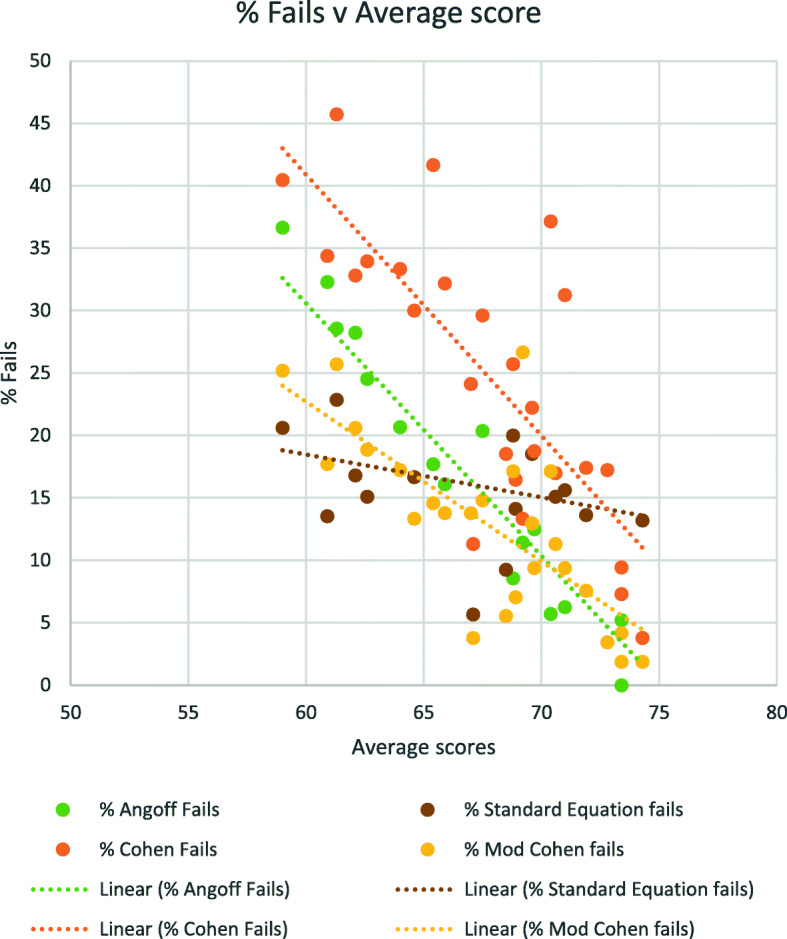
The percentage fail rate for each of four different standard setting methods, plotted against the average score in that exam, for a total of 26 exams. The linear trendline for each has been added for clarity

These results were also plotted against the average score in each assessment, as shown in Fig. 2. By all four methods, there is a linear relationship between the average score in the test, and the percentage of candidates who fail – as is to be expected, the higher the average score, the fewer candidates fail. However, the exponential curve method gives a much more stable result: the slope is much shallower than those of the other three methods. This accords with the lower Standard Deviation for the exponential curve method, as shown in Table 4.

A reasonable question would be to ask if Facility is dependent on the proportion of Borderline candidates in the test. We modelled the impact of changing this proportion, and the impact on the Facility of the test as a whole was small: for instance, the difference in cohort Facility when 15% versus 35% of the cohort were classed as Borderline was 3%.

This suggests that the overall performance of a cohort of students may be relatively stable to changes in the proportions of Borderline candidates, a point which we will return to in the Discussion.

## Discussion

For a context in which candidates have already undertaken multiple assessments previously standard-set by some conventional means, repeating the approach described here is possible and relatively straightforward. Candidates can be classified as Borderline on the basis of their performance across all assessments, and the equivalent of Fig. 1 plotted. We predict that a curve of the same form, and with constant values close to that of the standard exponential curve will be observed.

### Ways of using the exponential curve

The exponential curve equation can be used as a rapid and inexpensive primary standard setting method, in the same settings as Cohen and modified Cohen methodologies are currently employed. The Facility of each item is calculated in most item-banking applications. These Facilities can be exported to a spreadsheet and the exponential equation copied into the adjacent cells. Using the ‘fill down’ command in Excel, this takes seconds to do. The average value of the exponential equation outcomes is the cut score for the test as a whole.

We believe that the exponential equation is preferable to both Cohen methodologies, because it is derived from the results of all items and all candidates, rather than the results of one candidate in the test. In addition, it is more stable to changes in the average score than either Cohen methodology, or even Angoff approaches.

Alternatively, it could be used in conjunction with Angoff methods, to adjust the cut score value of individual items where there is a major discrepancy between the pre-calculated Angoff value and the observed Facility.

Compared to the Angoff method itself, this method avoids the need for assembling an expert reference group, and the time-consuming and contentious process of estimating an Angoff value for every item in the test. It is very much less costly terms of staff time to carry out, and may bring significant opportunity cost benefits.

The method may be useful in standard-setting new kinds of items, such as Very Short Answer Items, which have been observed to have lower Facilities than MCQs [8], and where Angoff values calculated by the usual method may not be appropriate.

### Challenges to this approach

The key issue is the stability of the constants C and F under different conditions.

Two conditions must be met for C and F to be relatively stable. The first is that the variance in difficulty of the assessments should be greater than the variance in ability of the candidates. It has indeed been demonstrated for medical students that “test-difficulty is a major source of variation while cohort and education effects probably are minor” [9]. Similarly, Cohen-Schotenaus and van der Vleuten concluded that “the most probable cause (of pass mark variability) is variability in test difficulty across different tests, both within and across courses”. This may be due to the fact that medical students are highly selected at entry to be at the top end of the academic ability spectrum.

The second is that the proportion of Borderline candidates should be a relatively stable proportion of the cohort as a whole. Again, the highly selected nature of medical students suggests this is a reasonable expectation. In any case, we have observed that significant variations in the proportion of Borderline candidates bring about only small changes in cohort Facility.

As a consequence, it is not unreasonable to think that C and F may vary only within a narrow range. Facility of items in a test as a whole may well be the most important variable in medical exams as previous authors have indicated.

## Conclusions

This novel standard setting method offers an inexpensive and easy to implement alternative to exisitng methods, which takes acoount of all candidates and all items. It is more stable to changes in mean score in the exam than alternative methods.

## Data Availability

The anonymised datasets analysed during the current study are available from the corresponding author on reasonable request.
